# How welfare states influence online platform work in Europe

**DOI:** 10.1177/09589287251357463

**Published:** 2025-07-18

**Authors:** Juliana Chueri, Petter Törnberg

**Affiliations:** 1 1190 Vrije Universiteit Amsterdam, Netherlands; 2 ILLC, 1234 University of Amsterdam, Netherlands

**Keywords:** platform work, decommodification, labor market institutions, skills, welfare state

## Abstract

Digital labor platforms are reshaping global labor markets by enabling the transnational contracting of service workers. While the dominant perspective emphasizes market forces, predicting that lower-wage countries will dominate the supply side, this view overlooks the institutional context in which platform labor emerges. This paper advances the argument that national welfare institutions are key to shaping participation in the platform economy. We provide the first large-scale cross-national comparative analysis of platform labor, combining micro-level data from one of the world’s largest remote work platforms with country-level indicators from 26 European countries. In line with market expectations, we find that lower-wage countries supply most low-skilled labor, while higher-wage countries show a more balanced distribution between low- and high-skilled workers. Crucially, however, our analysis reveals that greater welfare state generosity is associated with lower levels of platform participation, especially in low-skilled occupations. We argue that platform labor cannot be understood solely as a function of technological change or wage differentials. It is also an expression of structural constraints: where social protections are weak, people are more likely to turn to precarious forms of online work.

## Introduction

Recent years have seen the emergence of a new form of labor: “gig” or “platform” labor, which involves workers providing services through online platforms, such as Uber, Deliveroo, Amazon MTurk or Upwork. A growing academic literature has sought to theorize the nature of this form of labor and understand its implications for workers. Scholars however diverge in their conclusions. One group takes an optimistic view. They emphasize the purportedly positive impacts of platform labor, portraying it as emancipatory and as enabling flexibility by allowing workers to become “their own boss” (e.g., Sundararajan, 2017). The platform economy is thus cast as a new form of self-employment that promotes entrepreneurship and expands opportunities for autonomy and flexible work. On the other side of the debate, critical scholars take a more skeptical stance. They describe the ostensible emancipation and flexibility of platform labor as a façade, concealing high levels of precarity, exploitation, low pay, and intense algorithmic control (Gandini, 2019; Rahman and Thelen, 2019). Unlike entrepreneurs, platform workers typically lack the freedom to set their prices or working hours, which are often dictated by platform algorithms (Ravenelle, 2017; Meijerink and Keegan, 2019). This critical scholarship furthermore highlights that platform labor accelerates the individualization of risks by exempting companies from social responsibility (Thelen, 2018; Rahman and Thelen, 2019).

An emerging third strand of scholarship seeks to develop a middle ground, adopting a more nuanced perspective. While it acknowledges that platform work represents a precaritization of labor relations, it resists treating it as a monolithic category. Platform work ranges from alienating and repetitive to stimulating and well-compensated (Dunn, 2020; Hoang et al., 2020; Kalleberg and Dunn, 2016; Wood et al., 2019). Crucially, the quality of platform labor varies substantially across country contexts. For instance, estimates from the Online Labour Index 2020 (Stephany et al., 2021) show that while most platform workers based in the United States were engaged in clerical or data entry jobs, the most common platform jobs in European countries were software development, technology, and creative work.

Current scholarship primarily explains this cross-country variation in terms of market forces and market failures. The online platform economy is said to constitute a transnational labor market in which workers compete globally, leading to a race-to-the-bottom and the dominance of low-income countries in the platform labor markets (Graham and Anwar, 2019; Törnberg, 2023). One implication is that the platform economy can offer relatively high-quality work for high-skilled workers from low-income countries, as they can charge competitive fees while still earning a comparatively high salary in local terms (Kässi and Lehdonvirta, 2018). Research however also suggest that this transnationalization is incomplete, as buyers tend to prefer workers from higher-income countries for more complex tasks (Hoang et al., 2020; Huws, 2016). This preference is driven by non-monetary factors such as cultural proximity (Van Slageren et al., 2022), and cultural capital (Beerepoot and Lambregts, 2015; Demirel et al., 2021; Galperin and Greppi, 2019).

While the literature has focused on labor costs to explain the cross-country variation in the composition of the platform economy, we argue that it overlooks a crucial dimension: the role of welfare institutions. Recent scholarship suggests that platforms adapt and respond to local institutional conditions, and must be studied in relation to national institutional contexts (Bonoli et al., 2025; Funke and Picot, 2021; Picot, 2022; Thelen, 2018; Vallas and Schor, 2020). However, despite these insights, research has yet to carry out a large-scale study to systematically examine the relationship between national institutions and the platform economy (see Wood et al., 2019).

In this study, we aim to fill this gap through an empirical analysis that examines online platform work as institutionally situated and shaped by national context. We ask: to what extent do national wage levels and welfare institutions explain cross-country variation in the size and skill composition of the online platform economy? As in previous studies (Braesemann et al., 2022; Drahokoupil and Piasna, 2019; Pigatto et al., 2017), we focus on a single platform to answer these questions. We choose Upwork.com as our empirical case, which is one of the largest online platforms globally, and one that is seen as offering a wide range of jobs – ranging from low-skilled tasks such as clerical work, to high-skilled professional work such as software engineering (Blyth et al., 2022; D’Cruz and Noronha, 2016; Gandini, 2019; Pongratz, 2018; Kalleberg and Dunn, 2016; Wood et al., 2019). We draw on our empirical findings to theorize and explain the substantial cross-country differences, and the relationship between platform labor and welfare state institutions.

Our analysis is based on individual-level data from all platform workers registered on Upwork.com and residing in one of 26 European countries^1^, combined with country-level variables. Methodologically, we employ negative binomial regression with countries’ random intercepts to examine how welfare and labor market institutions influence the scale and scope of online platform work in Europe.

We conclude that online platforms in Europe, such as *Upwork.com,* operate as a transnational market in which lower-wage countries dominate the supply of low-skill labor. In contrast, higher national wages are associated with lower overall participation in platform work and a more balanced distribution between low- and high-skilled workers. Our findings moreover show that welfare institution shape the platform economy: after controlling for individual and country-level factors, greater welfare generosity is significantly associated with a lower prevalence of low-skilled platform work. On theother hand, welfare generoity has no significant effect on the prevalence of high-skilled jobs. Drawing on these empirical results, we theorize how national institutions shape platform labor by determining the demand and supply of workers: supplying high-skilled workers, or functioning as a financial buffer for unprotected and underemployed. The paper thus contribute to advancing the study of platform work as ituated in local labormaret and welfare institutions, and shows empirically how the platform economy is shaped by them.

## Forces shaping the online platform economy

The academic literature has highlighted the capacity of labor platforms to exempt themselves from statutory obligations by exploiting gaps in national legislation. As a result of this emphasis, platforms have often been treated as operating in a transnational regulatory vacuum – detached from national regulation (Boeri et al., 2020; Rosenblat and Stark, 2016; Woodcock and Graham, 2020). In lieu of relevant regulation, scholars have framed online platform as being shaped solely by market forces. Observed exception to this rule has been explained by discriminatory behavior among buyers, favoring workers from certain nationalities, which explains why workers from the Global North perform better than would be expected from labor costs alone (Beerepoot and Lambregts, 2015; Demirel et al., 2021; Galperin and Greppi, 2019).

However, recent theoretical work increasingly suggests that platforms should rather be understood as deeply embedded in national institutions, as they adapt their business strategies in response to local contexts (Bonoli et al., 2025; Funke and Picot, 2021; Picot, 2022; Thalen, 2018; Valle and Schor 2020; Törnberg, 2023). Building on these ideas, we suggest that welfare institutions are also important in shaping the composition the platform labor market, in particular the prevalence of high and low-skilled jobs.

We will now turn to outlining both these two perspectives on the forces shaping the platform labor market – first the literature focusing on the role of market forces, and then on the role of welfare state institutions – to suggest that platform labor market is shaped by the interaction between the two, and develop a non-competing set of hypotheses.

### Market forces and online platform labor as a quasi-planetary labor market

Scholars have argued that the emergence of the online branch of the platform economy – where digitally transferable services such as writing, editing, translating, and programming tasks are transacted via internet-based platforms – marks the rise of “planetary labor market”, in which geographical and institutional distance no longer matters, allowing companies to contract workers globally (Graham and Anwar, 2019). This development can be understood as an extension of the transformations that impacted the manufacturing sector, now applied to the service sector, which has historically been shielded from outsourcing pressures due to its reliance on local labor markets. Scholars suggest that such transnationalization of labor creates downward competitive pressures on wages, working conditions, and regulatory standards, as workers in the high-income countries are increasingly compelled to compete with workers in low-income countries (Graham and Anwar, 2019; Törnberg, 2023). The competitiveness of workers from low-wage locations is further reinforced by platform features such as customer reviews, rating systems, and the possibility of uploading work samples, tools that have been shown to reduce prejudice against workers from countries perceived as having lower educational quality (Lehdonvirta et al., 2019).

Workers from low-income countries have competitive advantages in the globalized labor market, as they offer services at rates that are low by global standards, but still relatively high within their national economy. For workers in higher income countries, however, a global labor market introduces a direct competition with lower-cost workers from low and middle-income countries, forcing them to reduce their fees or risk being outcompeted (Kässi and Lehdonvirta, 2018). This dynamic suggests that online platform work may be relatively unattractive for high-skilled workers in high-income European countries, and therefore remain a residual sector in the economy.

However, other scholars have argued that the transnationalization of the platform labor economy is partial and incomplete, as buyers still preferably to hire workers from geographically proximate economies (Beerepoot & Lambregts, 2015; Galperin and Greppi, 2019; Huws, 2016; Van Slageren et al., 2022). Research has shown that geographic proximity and common language are important factors in predicting transactions in the online platform economy (Van Slageren et al., 2022), indicating a degree of competitive protection to workers in high-wage economies, especially in more complex tasks (Hoang et al., 2020; Huws, 2016). Studies have furthermore suggested that geographic hierarchy, determined by perceived cultural capital and country reputation, plays a central role in workers' ability to obtain high ratings on platforms (Demirel et al., 2021; Sutherland et al., 2020). As a result, workers from high-income countries tend to perform better in the global platform labor market than the planetary labor market hypothesis would suggest (Beerepoot and Lambregts, 2015; Demirel et al., 2021; Galperin and Greppi, 2019).

The partial nature of the transnationalization of the platform labor market is reflected in the size of the European platform labor market. Despite the relatively high wage levels of European workers, data from the Online Labor Index 2020 (Stephany et al., 2021), based on the traffic of online platform work transactions across the five most important English-speaking platforms^2^, reveal that 16% of online platform workers in 2022 were based in Europe^3^, most of them working in software development and technology. This figure is far from negligible, especially considering that the European population represents 7.6% of the world population.

Following the literature that shows that the online platforms allow for a transnationalization of the labor market, we antecipate countries with lower wage levels to hold competitive advantages. As a consequence, we expect a grater presence of workers from countries with lower wages in both in high and low-skilled segments of the online labor market.

Hypothesis 1 Lower national wage levels are associated with a larger high-skilled platform labor market.

Hypothesis 2 Lower national wage levels are associated with a larger low-skilled platform labor market.

In line with literature suggesting that the transnationalization of the online platform market is incomplete, we expect that high-skilled platform workers will have relatively more opportunities in countries with higher national wages. This means that the skill composition of the platform economy will differ across countries, with a comparative higher share of high-skilled platform workers in countries with higher wages.

Hypothesis 3 Higher national wages are associated with a larger share of high-skilled platform labor.

### European welfare states and incentives to participate in the platform economy

Welfare state institutions constitute the second category of factors influencing the size and composition of the platfrom labor market. These institutions shape incentives for participation in the platform economy and play a crucial role in determining the availability of workers who are willing and able to engage in such labor.

This highlights the open question of *how* welfare institutions influence the availability of platform workers. When considering this question, it is necessary rely on previous literature examining the motivation of workers to engage in platform economy. While existing studies highlight heterogenous experiences of precarity of worker across platforms and occupations (Schor et al., 2020), we build on a body of literature that emphasizes fundamental differences in the motivations of high- and low-skilled platform workers. High-skilled workers’ decision to participate in platform work is often motivated by its flexibility, the opportunity to earn supplemental income, gain work experience and develop new skills (Hoang et al., 2020; Joyce et al., 2019; Ravenelle, 2019; Wood et al., 2019; Woodcock and Graham, 2020). Workers who engage in low-skilled platforms work — such as delivery, cleaning, caretaking, data entry and photo tagging — are typically driven by necessity, as a mean to cope with financial insecurity. (Dunn, 2020; Hoang et al., 2020; Joyce et al., 2019; Ravanelle, 2017; Van Doorn, 2017). These workers are often at the margins of the labor market, with many having experienced long periods of unemployment (Joyce et al., 2019).

The fact that low-skilled platform workers often turn this form of employment as a buffer for income insecurity suggests that platform labor can, at least in part, be understood as compensating for the absence or insufficiency of welfare support. Consistent with this interpretation, the literature has identified an association between insufficient welfare state protection and engagement in precarious work — characterized by low pay, low skill requirements, and temporary or insecure employment (Kalleberg, 2018; Lohmann, 2009). Comparative welfare state scholarship has documented significant differences in welfare generosity across European countries and between policy domains such as unemployment benefits, health care, social assistance, and family policies. In general, Northern European countries tend to provide more comprehensive support than Eastern and Southern European countries (Israel and Spannagel, 2019; Nelson, 2013; Scruggs and Ramalho Tafoya, 2022; Wang and Van Vliet, 2016).

While the division between Northern Europe and Southern/Eastern Europe provides a useful geographical framework for understanding variations in welfare generosity and comprehensiveness, it is important to recognize that many well-developed European welfare states have undergone shifts toward commodification (Scruggs and Ramalho Tafoya, 2022). These shifts have been motivated by a policy orientation that prioritizes social inclusion through labor market participation rather than a solely protective welfare state (Bonoli, 1997). This transition has been accompanied by the implementation of activation measures, which aim to reintegrate welfare recipients to the labor market, often under the guiding principle that “work should pay” (Immervoll, 2012; Rubery et al., 2018). As a result, even in countries that have historically been characterized by well-developed welfare states, precarious work has increasingly become the only available option for many individuals to make ends meet. 

Based on this, we expect welfare institutions to shape both the size and composition of the platform labor market. Specifically, lower welfare state generosity is likely associated with a greater supply of platform workers, particularly in low-skilled roles, as individuals facing income deprivation may see platform work as one of the few viable options for sustaining themselves financially.

We furthermore expect labor market institutions to play a role in shaping the prevalence of platform work. In recent years, many European countries have undergone labor market liberalization, resulting in increased permissiveness toward atypical forms of employment (Hipp et al., 2015). The prevalence of atypical employment shapes the platform labor market in two meaningful ways. First, atypical workers are less protected by welfare benefits (Jara Tamayo and Tumino, 2021), which makes them more likely to turn to the platform economy to secure financial stability. Given their limited access to employment-related benefits, atypical workers may perceive fewer distinctions between platform labor and non-platform work, making platform jobs relatively more attractive also to high-skilled workers.

Second, research indicates that platform labor in Europe should be viewed as complementary to other forms of atypical and part-time employment (Huws et al., 2018), meaning that platform work expands the range of options available for atypical workers to supplement their income. Consequently, and in line with prior studies (Cahuc and Postel-Vinay, 2002; Funke and Picot, 2021), we expect that labor market institutions more permissive toward atypical employment will increase the pool of platform workers. Considering this discussion, we develop another set of hypotheses:

Hypothesis 4 Less generous welfare states will be associated with a larger participation in low-skilled platform labor.

Hypothesis 5 A higher proportion of atypical labor will be associated with a larger participation in low-skilled platform jobs.

Hypothesis 6 A higher proportion of atypical labor will be associated with a larger participation in high-skilled platform jobs.

## Case selection and methodology

Our study focuses on European platform workers, as Europe provides a productive context for comparative research on the role of welfare institutions in shaping the platform economy. While workers in Europe generally benefit from higher income levels and welfare protection compared to global standards, there is sufficient variation to draw inferences about the impact of welfare institutions on the platform labor market. We study platform workers engaging in online work at *Upwork.com,* which is among the world’s largest online platforms (Kässi and Lehdonvirta, 2018). Although Upwork’s business model focuses on attracting high-skilled workers (Pongratz, 2018; Popiel, 2017), the literature highlights that the platform offers a wide range of jobs requiring varying skill levels (Blyth et al., 2022; Gandini, 2019). *Upwork.com* therefore provides a unique case for studying how institutions shape quantity and scope of platform work in each context.

Our study relies on data gathered from *Upwork.com* using a custom-written web scraper, collecting publicly available and anonymized information on all workers that are signed up on the platform and have declared in their profile to live in Europe^4^. The collected data is not considered personal data, as it cannot be connected to any individual; it pertrains solely to the amount and type of labor that has been carried out in the platform. The study follows the ethical guidelines for internet research provided by the British Sociological Association (BSA, 2017) and the Association of Internet Researchers (Franzke et al., 2020), and has been ethically approved by an institutional review board.

The data was collected in two steps. First, we first used the platform’s internal API to acquire the number of workers of different categories were registered – per country, per labor category, and based on how many hours they had worked. We found that the great majority of registered users on the platform had not worked a single hour during the last 6 months (see Supplemental Material for a detailed information on hours worked per country). In the selected countries, only 11,503 users out of 111,730 registered users were active. Considering the high percentage of workers that are registered on the platform but are not able or willing to obtain work, we focus our analysis exclusively platform workers who have worked at least 1 hour on the platform during the 6 months preceding the data collection (November/2022). In the second step, the full data on these active users were collected. We collected the following information of workers: declared occupation, whether the worker was recognized as “top talent” by the platform, whether they were affiliated with an agency, and the number of work samples available in their profile. Our dependent variable is the total number of hours each individuals worked through the platform.

### Individual level variables

We assess platform workers’ occupations by a matching between their employment titles^5^ and the ILO major occupation groups (professional, technician, service or sales worker, and clerical worker)^6^ (see Supplemental Material for details of this matching process). If the platform workers’ job title belongs to more than one major group, such as “*| Data Entry | Internet Research | Article Writing | Graphic Design”*, which falls on both clerical and professional work, we classify the platform workers’ occupation as the one that requires the lowest skill. This apporach assumes that workers listing a broad range of tasks are likely not highly specialized and primarily engage in low-skilled work. To test the robustness of this approach, we also conducted analyses using the inverse operationalization, in which workers with job titles spanning multiple categories were assigned to the highest corresponding skill level. These alternative analyses, detailed in the Supplemental Material, reveal result differences in the expected direction and do not alter the interpretations presented in the main text. If the platform worker does not provide a job title, or if the job title does not match any occupation, such as “200+ jobs finished with a perfect 5 stars rating and amazing reviews”, the occupation is classified as “other.” Following ILO classification, service, sales, and clerical occupations correspond to a skill level two (low-skill), whereas professionals and technical ocuppations correspond to skill levels three or four (high-skill).

“Top talent” is an accreditation given by *Upwork.com* to their best platform workers, based on clients’ evaluation and activity in the platform (Meijerink et al., 2024), serving as an important signal for potential new clients. Participation in agencies and work samples may also give credibility to the platform worker.

As our databases are anonymized, we cannot access gender, ethnicity, or any other individual-level information of the platform worker.

### Country level variables

Welfare generosity is assessed using the decommodification index provided by Israel and Spannagel (2019), which evaluates the generosity and coverage of three policies—unemployment benefits, healthcare, and social assistance. We prefer this measure over other comparative indicators of welfare generosity, such as the Comparative Welfare Entitlements Dataset developed by Scruggs et al. (2017), due to its broader country coverage, which includes Eastern European countries. As a robustness check, we repeat the analyses using a measure of welfare effort, operationalized as public social expenditure excluding pensions as a share of gross domestic product. We recognize the limitations of this variable in fully capturing the specific characteristics of welfare institutions and the actual protection provided to citizens; however, we follow the literature in continuing to treat it as a useful proxy for generosity (Jensen and Thompson, 2020). These additional analyses are available in the Supplemental Material and corroborate the findings presented in the main text.

The share of atypical workers is operationalized as the percentage of individuals who declared to engage involuntarily in temporary employment, as voluntary temporary employment is more likely to represent a life choice rather than labor market constraints. We measure national wage levels using the average hourly national wage in U.S. dollars. This variable is also included as an alternative explanatory variable in our models, as lower average national wages are expected to create stronger incentives for participation in the online platform economy, where income from platforms may be comparatively more attractive than earnings in the local job market. Finally, we control for the unemployment rate, anticipating that difficulties in securing traditional employment may drive workers to seek opportunities within this sector.

Table 1 summarizes the individual and country-level variables of this study.

**Table 1.** Variables of the study.

Variable level	Variable	Explanation	Source
Individual	Hours worked in the platform	Total of hours work in the platform	Upwork
Individual	Occupation	Declared occupation	Upwork
Individual	Top talent	Platform classification	Upwork
Individual	Agency	Participation in an agency	Upwork
Individual	Total of portfolio items	Number of work samples in the portfolio	Upwork
Individual	Country of work	Declared location	Upwork
Country	Decommodification	Decommodification index	Israel and Spannagel (2019)
Country	National salary	Average national hourly salary by occupation	ILO
Country	Unemployment ratio	Percentage of unemployment among the working force	Eurostat
Country	Public social welfare expenditure	Public social welfare expenditure as GDP ratio excluding pensions	Eurostat
Country	Risk of poverty and social exclusion	Percentage of the population at risk of poverty and social exclusion	Eurostat
Country	Temporary employment	Percentage of workers with involuntary temporary contracts	Eurostat

We conduct a two-step analysis. The first step takes a descriptive approach, illustrating the participation of platform workers by country and occupation. The second step relies on a multilevel analysis with individual data and country random intercepts. We estimate models to evaluate the factor that determine the total number of hours an individual works on the platform. As we have a highly disperse count dependent variable (refer to Table S1 in the Supplemental Material for the variable distributions), we employ negative binomial regressions.

## Results

### Descriptive statistics

Table S1 in the Supplemental Material reveals that 77% of platform workers report performing high-skilled work, which aligns with the platform strategy of attracting high-skilled professionals (Pongratz, 2018). This ratio is moreover consistent with estimates from the Online Labor Index regarding the skill composition of platform workers in Europe (Stephany et al., 2021), based on a sample from the five largest online platforms. Taken togheter these findings suggest that the results of this study are representative of broader trends across European online platforms.

**Figure 1.**
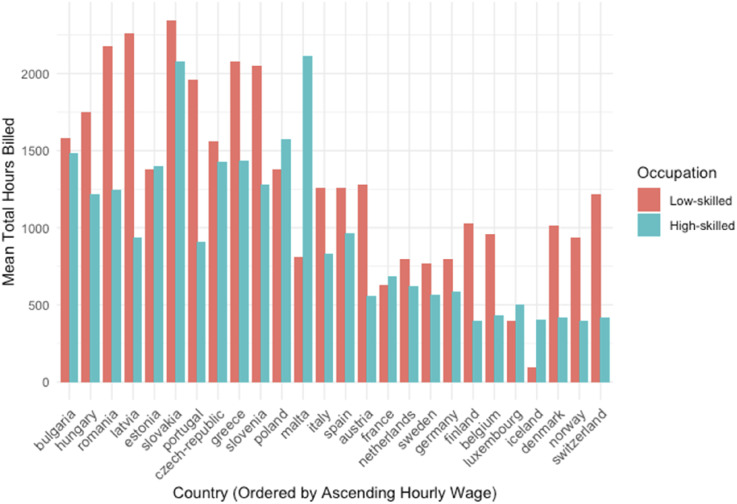
Average hours of work accumulated in the platform, by occupation skills, in low- and high-skilled occupations by country, ordered by average hourly salary.

Figure 1 displays the average number of hours worked by low- and high-skilled platform workers in each of the countries analyzed. The countries are arranged in ascending order based on their average wage. Although the vast majority of platform workers report engaging in high-skilled occupations, the figure shows that, in all countries except Estonia, Iceland, Luxembourg, and Malta, low-skilled workers accumulate more hours on the platform. This finding supports our claim that Upwork.com functions as a marketplace for a wide range of jobs and is therefore well-suited for studying the macro-level determinants of the size and composition of national platform economies.

Although there are substantial country variations, Figure 1 reveals a negative relationship between local wages and hours worked on the platform: workers in higher-wage countries tend to work fewer hours on the platform. The effect of national wages on skill composition is, however, less clear-cut. In line with our expectations, countries with relatively high wages, such as France, the Netherlands, Sweden, and Germany, exhibit a more balanced participation of low- and high-skilled platform workers. By contrast, countries with very high salaries, such as Norway and Switzerland, exhibit a clear predominance of low-skilled platform workers.

### Inferential statistics

To test our expectations regarding differences in participation in the online platform economy across Europe, we employ negative binomial regression with countries’ random intercepts, taking the total hours worked by individuals on the platform as the dependent variable. We start our inferential analysis by investigating the relationship between national average salaries and the skill composition and size of the platform. The first model incorporates individual-level controls: the award of a ‘top talent’ badge on the platform, number of portfolio items, agency affiliation, and the skill level. Country-level variables, such as the unemployment rate and average wage, were also included. Finally, an interaction effect between workers’ skill level and the national average wage is included. The full regression output is available in the Supplemental Material.

**Figure 2.**
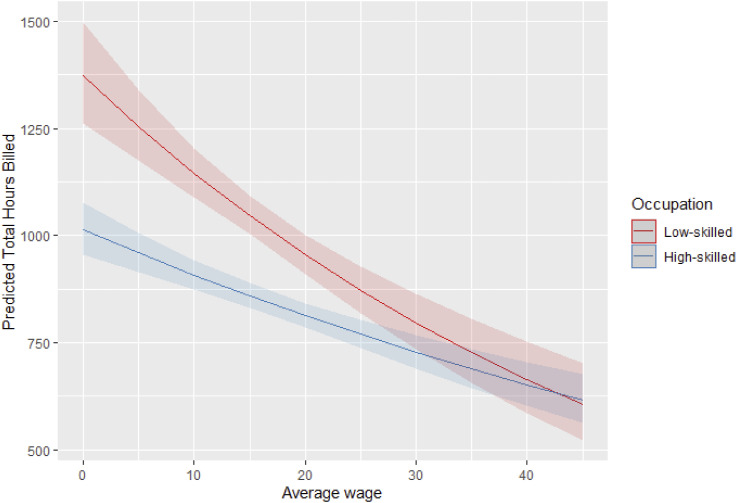
Interaction between national average hourly wages (US$) and workers’ skill on the predicted hours of work on the platform. 95% C.I.

Figure 2 plots the effect of the interaction between skill levels and national hourly wages on the predicted average number of hours worked in the platform. While most active workers on the platform report performing high-skilled tasks, low-skilled workers tend to work more hours on the platform. In line with hypotheses 1 and 2, the analysis shows that individuals in countries with lower average national wages work, on average, more hours on the platform, both in low- and high-skilled occupations. In line with hypothesis 3, which states that higher national wages are associated with a larger share of high-skilled platform workers, the figure shows that in countries where the average wage exceeds $25/hour, the share of high- and low-skilled platform workers becomes statistically equivalent.

**Figure 3.**
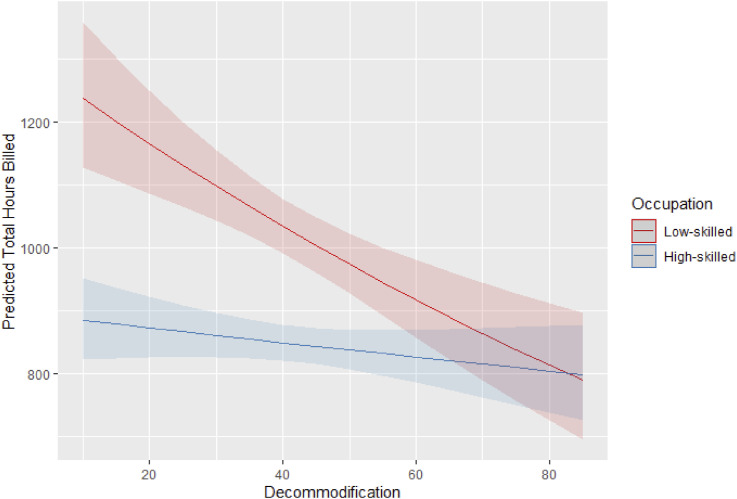
Interaction between decommodification index and workers’ skill on the predicted hours of work on the platform. 95% C.I.

We next explore the impact of welfare institutions on the size and skill composition of the platform, using the same model specification as before but replacing the previous interaction term with one between workers’ skill level and the country’ welfare decommodification index (refer to the Supplemental Material for the full regression output). Figure 3 illustrates this relationship by showing the predicted average hours worked by low- and high-skilled workers across varying levels of decommodification. In line with Hypothesis 4, our findings indicate that higher decommodification is associated with a lower participation in low-skilled platform work, while it does not significantly afect the number of hours worked in high-skill jobs. As a result, in countries where decommodification index exceeds 58, the predicted hours worked by high- and low-skilled workers are statistically equivalent.

**Figure 4.**
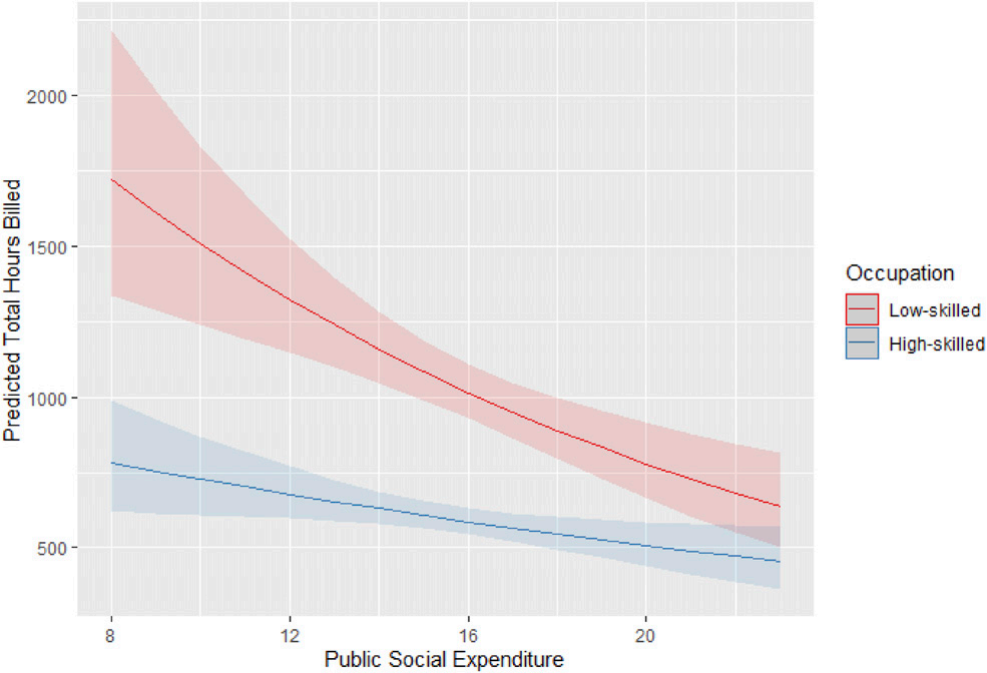
Interaction between social public expenditure as GDP ratio and workers’ skill on the predicted hours of work on the platform. 95% C.I.

To check for robustness of our findings, we also use an alternative measure of welfare generosity: welfare effort measured as the public social expenditure without pension as a GDP ratio (refer to the Supplemental Material for the full regression output). Figure 4 illustrates this relationship by showing the predicted average hours worked by low- and high-skilled workers across varying levels of public social expenditure.

In line with the results presented in Figure 3, higher welfare expenditure is associated with lower participation in low-skilled platform work. Welfare effort however has only a modest negative effect on the participation of high-skill platform workers. Consequently, in countries where social expenditure, excluding pensions, exceeds 21% of GDP, the predicted hours worked by high- and low skilled-workers are statistically comparable. 

As our variables for welfare generosity encompass a wide range of social programs, we conducted further analyses to investigate wheter participation in the platform is particularly driven by inadequate safety-net provisions. We thus revisited the previous analysis, incorporating an interaction between the percentage of the population at risk of poverty or social exclusion and workers’ skill levels. The results, available in the Supplemental Material, reveal a strong association between a higher proportion of the population at risk of poverty or social exclusion and a greater number of hours in low-skilled platform work. In contrast, higher levels of poverty and social exclusion risks do not have a statistically significant effect on the hours worked in high-skilled platform jobs. This additional analysis confirms that participation in low-skilled platform work is particularly linked to social vulnerability and inadequate public provision of social safety nets.

**Figure 5.**
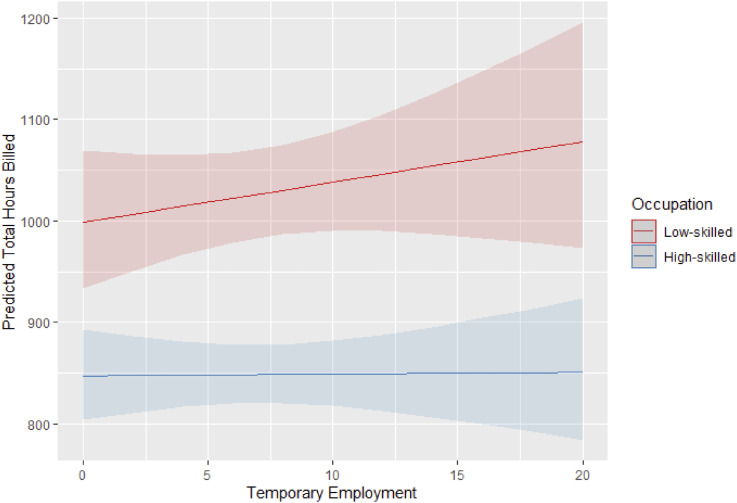
*Interaction between* the ratio of involuntary temporary employment *and workers’ skill* on the predicted hours of work on the platform. 95% C.I.

We finally turn to the analysis of how labor market institutions are associated with size and skill composition of the platform. We repeat the model specifications used in the previous analyses, now including an interaction between the percentage of the national population engaged in involuntary temporary employment and platform workers’ skill levels (refer to the Supplemental Material for the full regression output). Figure 5 illustrates how varying levels of involuntary temporary employment relates to the accumulated hours in the platform of low and high-skilled worker.

The figure shows that in countries with a higher percentage of involuntary temporary employment, low-skilled workers accumulate, on average, more hours on the platform. However, this result is not statistically significant at the 95% confidence level, we therefore do not accept Hypothesis 5. Contrary to our expectations, the presence of involuntary temporary contracts does not significantly impact the supply of workers for high-skilled platform labor, leading us to reject Hypothesis 6.

We repeated this analysis using the share of workers on very short-term contracts, defined as contracts lasting up to three months. The results, available in the Supplemental Material, indicate that in countries with a higher prevalence of short-term employment, low-skilled workers accumulate more hours on the platform. In contrast, the predicted hours worked by high-skilled platform workers are not affected by this country-level variable. This finding suggests that it is the prevalence of extreme employment insecurity, rather than atypical employment more brodly, that drives participation in low-skilled platform work.

## Discussion and conclusion

Research on the online platform economy typically relies on market forces to explain cross-country variations in the composition of the online platform labor market. According to this perspective, the online platform economy can be seen as a transnational labor market where workers from around the world compete. Consequently, a North-South divide in the composition on online platform labor is expected: workers living in lower wages countries should be expected to dominate the supply, as they can charge competitive rates while still earning relatively high incomes compared to local salaries. The existence of high-skilled platform workers in high-wage economies, on the other hand, is explained by cultural factors or market failures, such as buyers’ discriminatory behavior.

Recent theoretical work has however called attention to the role of institutional context in shaping the platforms economy (Bonoli et al., 2025; Funke and Picot, 2021; Picot, 2022; Thelen, 2018; Vallas and Schor, 2020). Despite of this, no research has yet systematically examined how welfare institutions impact the size and the predominance of difference types of jobs in online platform. This study has aimed to address this research gap and contribute to the advancement of platform economy studies by examining platform work as shaped by the national institutional context. We conduct a comparative analysis to investigate how welfare and labor market institutions influence cross-country variations in the size and skill composition of the online platform economy.

The study has relied on a comprehensive dataset encompassing individual-level data of all workers registered on the online platform *Upwork.com* in 26 European countries. We employed multilevel negative binomial regressions with countries’ random intercepts to study how individual and country-level data influence the total hours of work in the platform. Our findings confirmed that the platform functions as a transnational marketplace at the European level, with workers from European countries with lower salaries dominating the labor supply. Individuals living in countries with higher wages accumulate less hours in the platform, and workers engaging in low-skilled and high-skilled jobs work similar numbers of hours in the platform. However, the results also shed light on how the platform economy interacts with national institutions, showing that welfare institutions are associated with differentiated skill composition and levels of participation in the platform. We found that welfare generosity has a significant effect in reducing the participation of workers in low-skilled platform labor. On the other hand, it does not alter the hours worked in high-skill platform jobs.

These findings support previous studies suggesting that, for some workers, platform work functions as a substitute for insufficient welfare provision, such as unemployment benefits and childcare services. Specifically, we found that higher shares of the population at risk of poverty and social exclusion are associated with greater participation in low-skilled platform work. This suggests that, for some individuals, platform labor may function as an alternative to an adequate safety net—a means of making ends meet in the absence of other forms of support. In contrast, our results show that welfare generosity does not significantly affect participation among high-skilled platform workers. This aligns with previous studies indicating that high-skilled workers are often motivated by the flexibility of platform work or the opportunity to earn supplemental income, rather than economic necessity. 

Contrary to our expectation, labor market liberalization, operationalized as the prevalence of involuntary temporary employment, is not significantly associated with platform labor participation, for either low- or high-skilled workers. Exploring the relationship between national labor market institutions and platform labor thus remains an important avenue for future research.

Our conclusion highlights the need to examine the platform economy within the broader context of national institutions. Platform labor is embedded in welfare and labor market structures and cannot be understood as a separate, decontextualized phenomenon. Its growth and consequences are fundamentally shaped by national systems of welfare provision, labor regulation, and economic policy. This perspective challenges narratives that frame the rise of platform work primarily as a product of technological innovation. Instead, it situates platform labor within longer-term processes of welfare retrenchment and labor market liberalization, which have contributed to the growing commodification of labor. Low-skilled platform work, in particular, depends on a supply of workers insufficiently protected by welfare institutions—and may itself reinforce commodification by serving as a substitute for public support systems. 

As a result, understanding worker participation in platform labor, its societal implications, and how it can be effectively regulated requires close attention to national welfare and labor market institutions. While targeted regulatory initiatives—such as the European Union’s Platform Work Directive—are essential for improving working conditions, they are not sufficient on their own. Ensuring adequate welfare provision for all categories of workers is equally critical, particularly in addressing the structural drivers behind the growth of low-skilled platform work across Europe.

This study offers an initial empirical step toward incorporating national institutions into the analysis of platform labor, through a systematic cross-national comparison of platform workers across European countries. Future research should build on this foundation by conducting more in-depth analyses of the dynamic interplay between digital transformation and national institutions, as well as comparative studies of how different institutional contexts shape participation in the platform economy.

Some limitations of this study should be mentioned. Our analyses are based on data from a single platform, and participation may thus be influenced by the popularity of the specific platform in the country. However, *Upwork.com* is one of the most important platforms in the world, and one of the few that offer both high and low-skilled jobs. Moreover, a comparison between our data and Online Labour Index 2020 shows that the composition of the *Upwork*.com worker force in terms of skills is similar to the European platform labor market as a whole. In a field that lacks representative data on platform workers, this study represents the first step to studying how context influences the dynamics of the European online platform economy. Future research may seek to expand the study to more countries and include participation in a broader range of online platforms.

## Supplemental Material

Supplemental Material - How welfare states influence online platform work in Europe Supplemental Material for How welfare states influence online platform work in Europe by Juliana Chueri and Petter Törnberg in Journal of European Social Policy

## Notes

1. Our sample includes workers from Austria, Belgium, Bulgaria, Czechia, Denmark, Estonia, Finland, France, Germany, Greece, Hungary, Iceland, Italy, Latvia, Luxembourg, Malta, the Netherlands, Norway, Poland, Portugal, Romania, Slovakia, Slovenia, Spain, Sweden, and Switzerland. We included all countries for which the decommodification index was provided by Israel and Spannagel (2019). We excluded the UK and Ireland from the sample as they were outliers in terms of participation, due to advantages related to the platform’s working language being English.

2. Upwork, Freelance.com, Guru.com. Mturk.com. Peopleperhour.com.

3. Author estimation based on OLI data accounting for workers declaring to be based in Albania, Austria, Belarus, Belgium, Bosnia and Herzegovina, Bulgaria, Croatia, Cyprus, Czech Republic, Denmark, Estonia, Finland, France, Germany, Greece, Hungary, Iceland, Ireland, Italy, Latvia, Lithuania, Luxembourg, Macedonia, Malta, Moldova, Monaco, Netherlands, Norway, Poland, Portugal, Romania, Serbia, Slovakia, Slovenia, Spain, Sweden, Switzerland, Turkey, Ukraine, or United Kingdom.

4. Data collection was concluded on 7 November 2022.

5. Self-declared by the platform worker.

6. This match proceeds in two steps. First, we conduct a textual match between gig workers self-declared occupation and the ISCO-08’s list of occupations. Second, we reduce those occupations into professional, technician, service or sales worker, and clerical worker.
