# Supplementary material for: How welfare states influence online platform work in Europe
Source: J Eur Soc Policy. 2025 Jul 18;36(2):119–35. doi: 10.1177/09589287251357463 (PMC13052987; doi:10.1177/09589287251357463)

Supplementary material: States and Platforms: How Welfare States Influence Online Platform Work in Europe

Figure S1 shows the number of platform workers registered in the platform by country, regardless of whether they performed any work. Participation varies greatly across Europe and only can be partially explained by countries' population size.

Figure S1. *Numbers of workers registered in upwork.com by country*

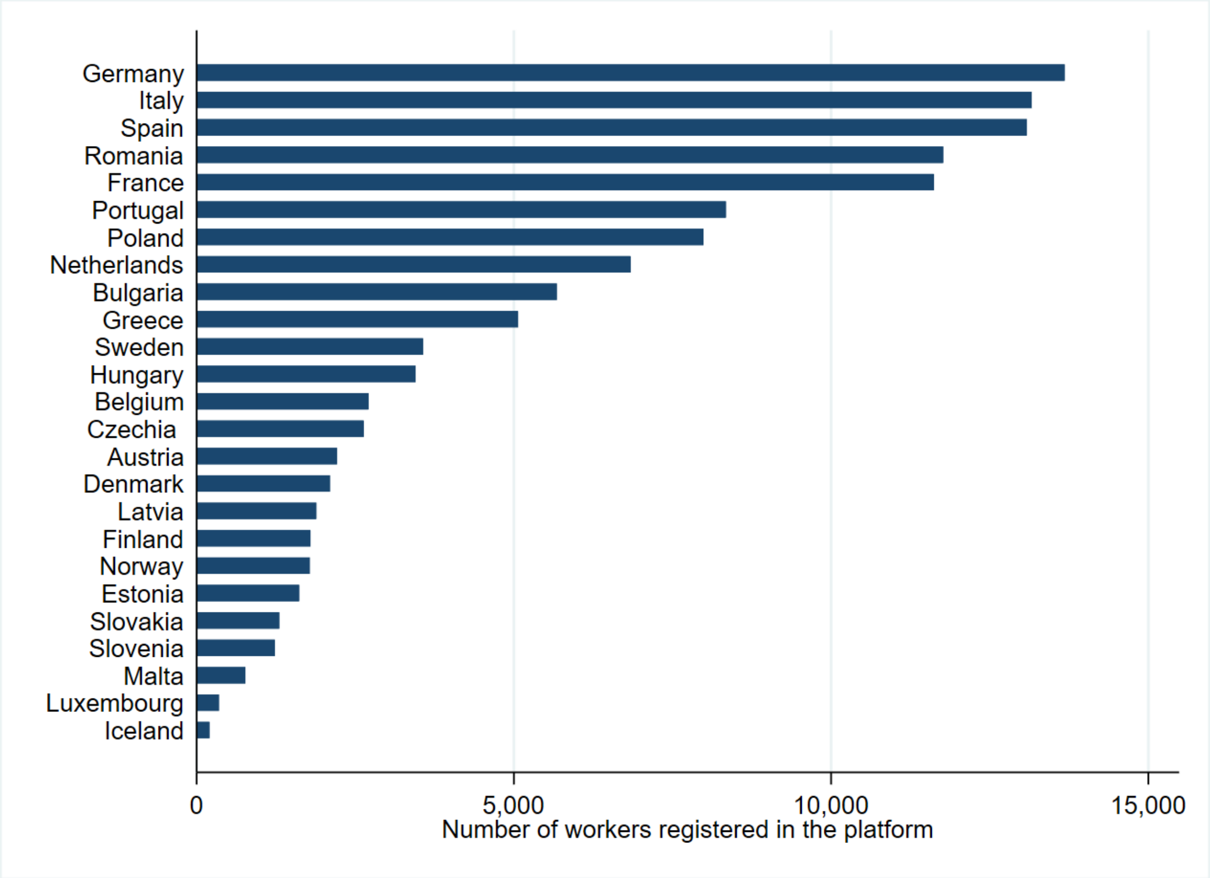

Figure S2 shows that the vast majority of workers registered on the platform did not work a single hour, and only 2.1% accumulated more than 1,000 hours of work on the platform. Therefore, we included only active workers in the analysis—those who were registered on the platform and performed at least one hour of work during this period. As a result, 89.7% of the 111,730 registered users were considered inactive. These workers were excluded from the empirical analysis, resulting in a sample of 11,503 active workers.

Figure S2. *Percentage of workers in each country who have accumulated zero, one, 100, or 1000 hours of work in the Upwork.com*

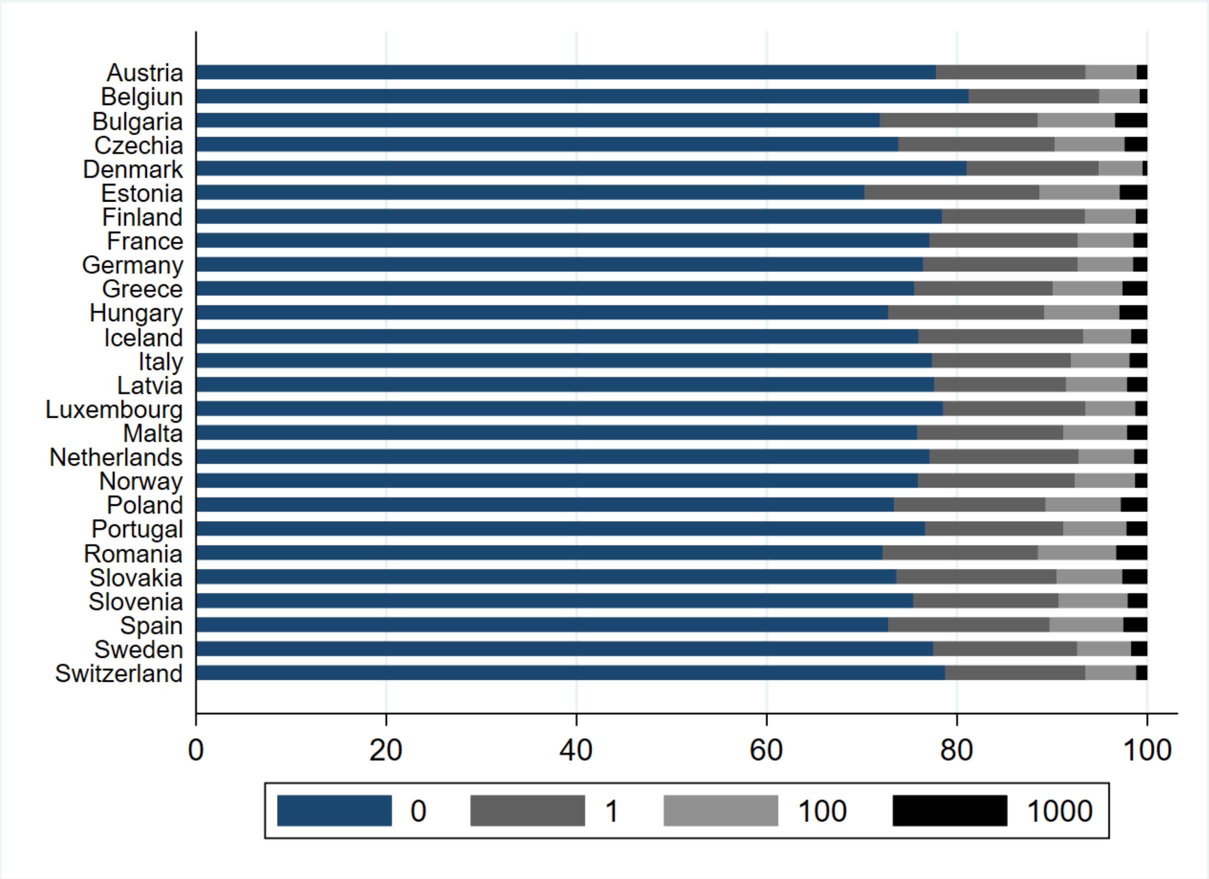

Table S1 summarizes the distribution of the variables used in the inferential analyses.

Table S1. *Summary of the distribution of the variables of the inferential analyses*

Variable	Number of observations	Mean	Standard deviation	Min	Max
Total hours billed	11503	1067.94	2648.63	0	66795.7
Top talent	11503	0.16	0.37	0	1
Occupation	11503	0.77	0.42	0	1
Agency	11503	0.1	0.29	0	1
Portfolio items	11503	4.25	10.13	0	364
Unemployment	11503	7.27	3.69	3.4	14.8
Average country wage	11503	16.28	7.86	3.62	41.93
Public social welfare expenditure	11503	16.04	4.44	8.9	23.2
Decommodification index	11503	41.87	15.57	14.88	80.62
Temporary employment	11484	7.32	6.02	0.3	19.5
Precarious employment	11503	1.4	1.22	0	4.9
Risk of poverty	11484	17.4	3.86	9.5	23.8

Classification of profiles according to occupations

The classification of profiles according to professions was established using a dictionary. This dictionary was formulated based on the 100 most frequently occurring words occurring in platform workers' occupation descriptions. These words were meticulously categorized by the main investigators according to the International Standard Classification of Occupations (ISCO-08), into the major occupational groups whenever they unambiguously pertained to a specific occupation. Words that did not pertain to any occupation were left uncategorized. The list of 100 words and their respective classifications underwent a review process.

Subsequently, researchers matched profiles with occupations using python. Following this, researchers conducted a re-evaluation of profiles without predefined categorizations. During this final step, some profiles were manually classified if they clearly referenced one or more occupations.

Table S2 shows the total number of active platform workers by county and the percentage of workers in each occupation as a percentage of the total number of platform workers in our sample.

***Table S2**. Platform workers distributed by country and occupations.*

**Country**	**Low-skilled**	**High-skilled**	**Total**	**% of the total workers**
Austria	19%	81%	**189**	1.64%
Belgium	20%	80%	**201**	1.75%
Bulgaria	26%	74%	**551**	4.79%
Czechia	24%	76%	**269**	2.34%
Denmark	22%	78%	**161**	1.40%
Estonia	14%	86%	**183**	1.59%
Finland	20%	80%	**141**	1.23%
France	22%	78%	**972**	8.45%
Germany	22%	78%	**1202**	10.45%
Greece	28%	72%	**469**	4.08%
Hungary	31%	69%	**331**	2.88%
Iceland	16%	84%	**19**	0.17%
Italy	25%	75%	**1123**	9.76%
Latvia	26%	74%	**162**	1.41%
Luxembourg	10%	90%	**29**	0.25%
Malta	29%	71%	**62**	0.54%
Netherlands	22%	78%	**586**	5.09%
Norway	17%	83%	**146**	1.27%
Poland	15%	85%	**871**	7.57%
Portugal	24%	76%	**699**	6.08%
Romania	26%	74%	**1147**	9.97%
Slovakia	26%	74%	**131**	1.14%
Slovenia	29%	71%	**104**	0.90%
Spain	26%	74%	**1314**	11.42%
Sweden	19%	81%	**275**	2.39%
Switzerland	22%	78%	**166**	1.44%
**Total**	**2676**	**8827**	**11503**	100.00%
**% of the total workers**	23.26%	76.74%	100.00%	

Table S3 displays the regression outputs that originated the figures 2, 3, 4 and 5 in the main manuscript.

***Table S3**. Output of negative binomial regressions on the number of hours of work on the platform, with random country intercepts and interaction.*

	Model figure 2	Model figure 3	Model figure 4	Model figure 5
Intercept	6.931 ***	7.069 ***	7.842 ***	6.829 ***
	(0.049)	(0.063)	(0.198)	(0.043)
Top talent	1.313 ***	1.313 ***	1.888 ***	1.312 ***
	(0.021)	(0.021)	(0.039)	(0.021)
Occupation	-0.304 ***	-0.382 ***	-1.027 ***	-0.164 ***
	(0.045)	(0.055)	-0.127	-0.031
Average wage	-0.018 ***	-0.010 ***	-0.025 ***	-0.013 ***
	(0.002)	(0.002)	(0.007)	(0.001)
Agency	0.264 ***	0.265 ***	0.596 ***	0.263 ***
	(0.027)	(0.027)	(0.049)	(0.027)
Portfolio items	0.005 ***	0.005 ***	0.010 ***	0.005 ***
	(0.001)	(0.001)	(0.002)	(0.001)
Unemployment	0.005	0.002	0.018	0.004
	(0.003)	(0.004)	(0.011)	(0.005)
Occupation: Average wage	0.007 **			
	(0.003)			
Decommodification		-0.006 ***		
		(0.001)		
Occupation: Decommodification		0.005 ***		
		(0.001)		
Social expenditure			-0.066 ***	
			(0.016)	
Occupation: Social expenditure			0.030 ***	
			(0.008)	
Temporary employment				0.004
				(0.004)
Occupation: Temporary employment				-0.004
				(0.003)
AIC	165.962.963	165.952.266	165.075.869	165.735.939
Log Likelihood	-82.971.481	-82.965.133	-82.526.935	-82.856.970
Num. obs.	11503	11503	11503	11484
Num. groups : country	26	26	26	25
Var: country (Intercept)	0.001	0.001	0.021	0.001

*** $p < 0.001$; ** $p < 0.01$; * $p < 0.05$

Figures S3, S4, S5 and S6 repeat the analyses presented in Figures 2, 3, 4 and 5 of the main text, using an alternative categorization of occupation. In our sample, 756 individuals reported performing both low- and high-skilled occupations. In the original analyses, these individuals were classified as low-skilled workers. Below, figures S3, S4, S5 and S6 present analyses with an alternative classification, where these workers are classified as high-skilled

Figure S3. *Interaction between national average hourly wages (US$) and workers' skill on the predicted hours of work on the platform. 95% C.I.*

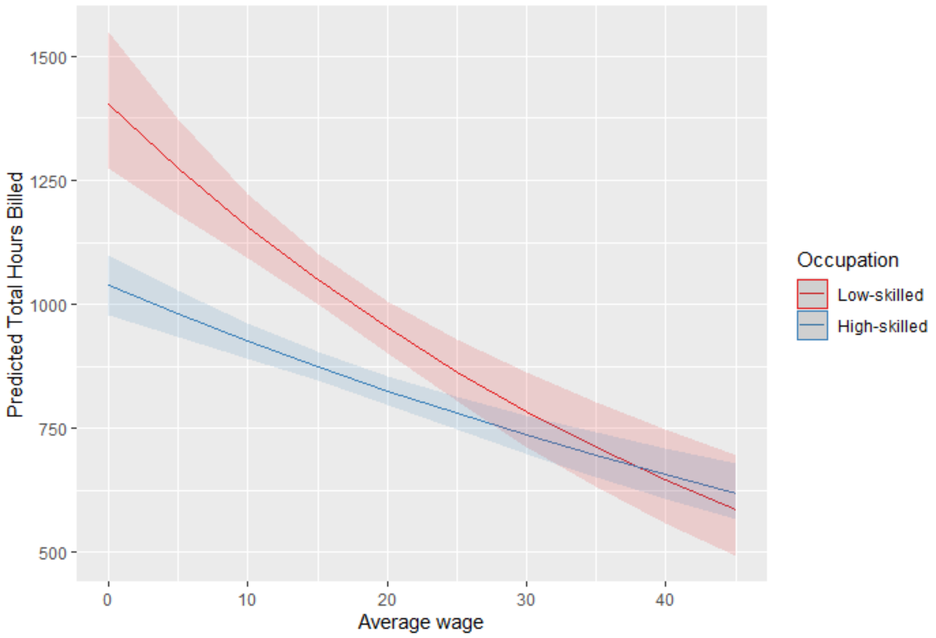

Figure S4. *Interaction between decommodification index and workers' skill on the predicted hours of work on the platform. 95% C.I.*

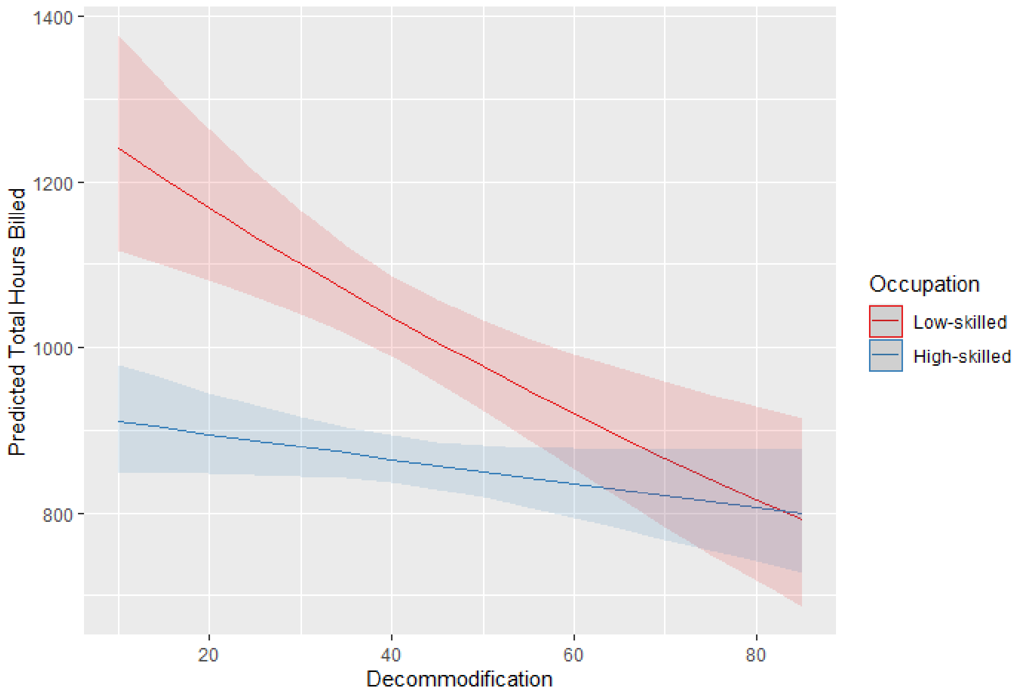

***Figure S5.** Interaction between social public expenditure as GDP ratio and workers' skill on the predicted hours of work on the platform. 95% C.I.*

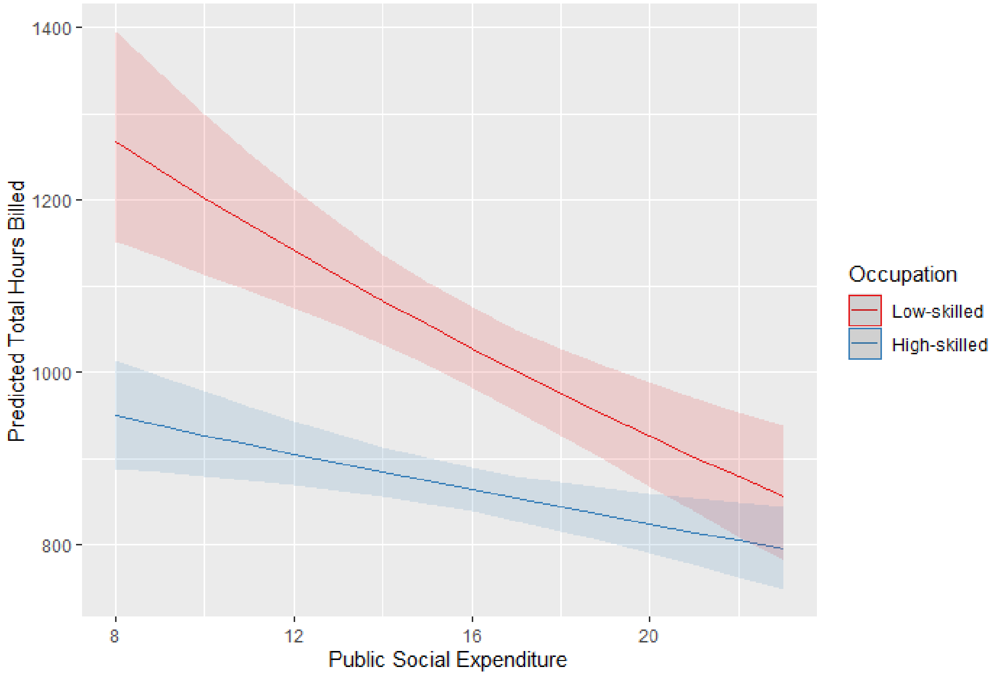

***Figure S6.** Interaction between the ratio of involuntary temporary employment and workers' skill on the predicted hours of work on the platform. 95% C.I.*

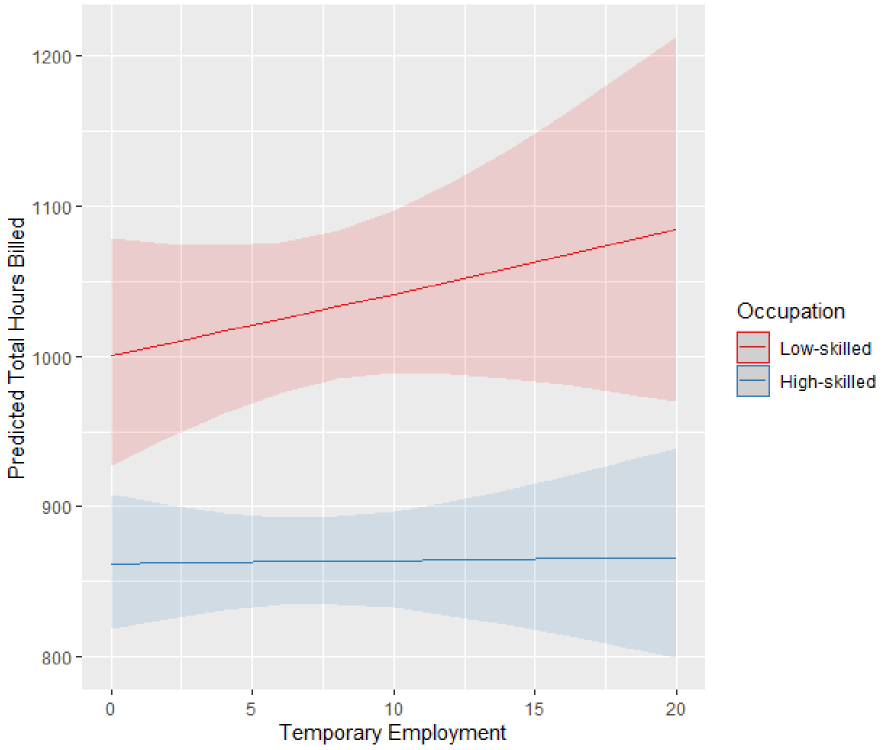

Figures S7 presents complementary analyses to figures 2 and 3 showing that risk of poverty is associated more supply of low-skilled workers in the platform.

Figure S7. *Interaction between the share of the population at risk of poverty and social exclusion and workers' skill on the predicted hours of work on the platform. 95% C.I.*

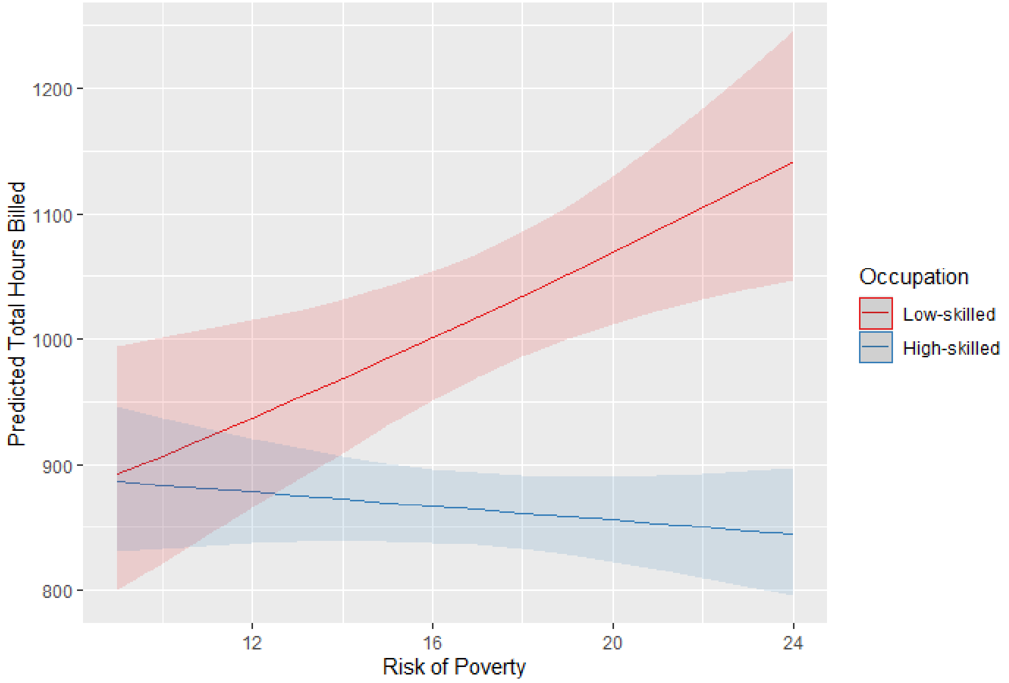

Figure S8 displays an alternative analysis to figure 6, using the percentage of workers in short-term contracts, up to three months, as the independent variable.

Figure S8. *Interaction between the ratio of short-term employment contracts and workers' skill on the predicted hours of work on the platform. 95% C.I.*

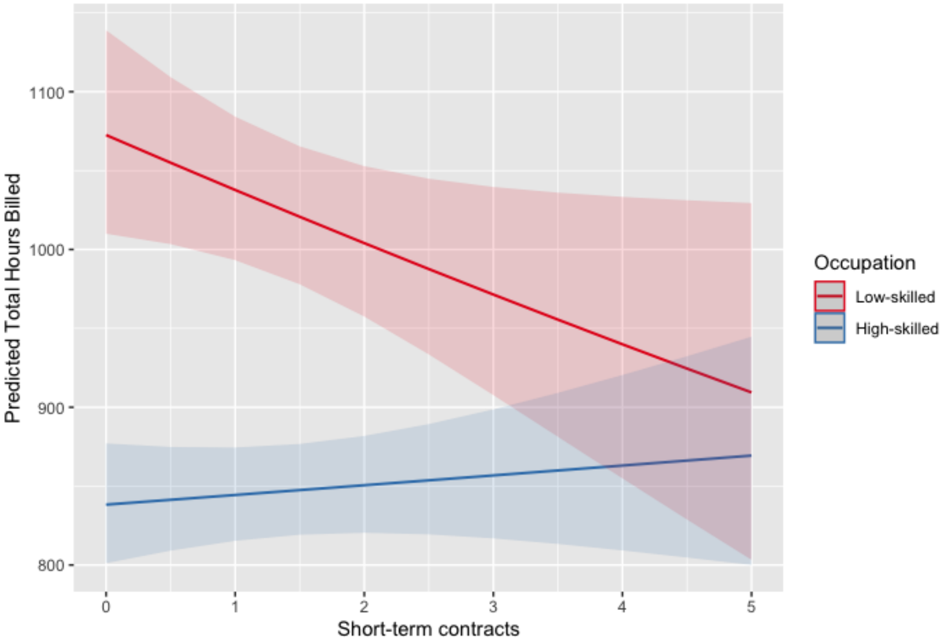

Supplement: Supplemental Material - How welfare states influence online platform work in Europe [file sj-pdf-1-esp-10.1177_09589287251357463.pdf]
